# Gel-forming antagonist provides a lasting effect on CGRP-induced vasodilation

**DOI:** 10.3389/fphar.2022.1040951

**Published:** 2022-12-08

**Authors:** Chia Lin Chang, Zheqing Cai, Sheau Yu Teddy Hsu

**Affiliations:** ^1^ Department of Obstetrics and Gynecology, Chang Gung Memorial Hospital Linkou Medical Center, Chang Gung University, Taoyuan, Taiwan; ^2^ CL Laboratory LLC, Gaithersburg, MD, United States; ^3^ Adepthera LLC, San Jose, CA, United States

**Keywords:** CGRP, adrenomedulin, self-assemble, migraine, CLR/RAMP, vasodilation, liquid gel, pharmocokinetics

## Abstract

Migraine affects ∼15% of the adult population, and the standard treatment includes the use of triptans, ergotamines, and analgesics. Recently, CGRP and its receptor, the CLR/RAMP1 receptor complex, have been targeted for migraine treatment due to their critical roles in mediating migraine headaches. The effort has led to the approval of several anti-CGRP antibodies for chronic migraine treatment. However, many patients still suffer continuous struggles with migraine, perhaps due to the limited ability of anti-CGRP therapeutics to fully reduce CGRP levels or reach target cells. An alternative anti-CGRP strategy may help address the medical need of patients who do not respond to existing therapeutics. By serendipity, we have recently found that several chimeric adrenomedullin/adrenomedullin 2 peptides are potent CLR/RAMP receptor antagonists and self-assemble to form liquid gels. Among these analogs, the ADE651 analog, which potently inhibits CLR/RAMP1 receptor signaling, forms gels at a 6–20% level. Screening of ADE651 variants indicated that residues at the junctional region of this chimeric peptide are important for gaining the gel-forming capability. Gel-formation significantly slowed the passage of ADE651 molecules through Centricon filters. Consistently, subcutaneous injection of ADE651 gel in rats led to the sustained presence of ADE651 in circulation for >1 week. In addition, analysis of vascular blood flow in rat hindlimbs showed ADE651 significantly reduces CGRP-induced vasodilation. Because gel-forming antagonists could have direct and sustained access to target cells, ADE651 and related antagonists for CLR/RAMP receptors may represent promising candidates for targeting CGRP- and/or adrenomedullin-mediated headaches in migraine patients.

## Introduction

Migraine is a neurovascular disorder characterized by episodic and unilateral headache with hypersensitivity to light, sound, and movement (Erdener and Dalkara, 2014; Schuster and Rapoport, 2016), and is the second most disabling disorder worldwide (Edvinsson et al., 2018; Kumar et al., 2022). One migraine treatment target that has attracted great attention is the calcitonin gene-related peptide (CGRP)-mediated CLR/RAMP1 receptor (i.e., the calcitonin receptor-like receptor (CLR) and receptor activity-modifying protein 1 (RAMP1) complex) signaling (Tso and Goadsby, 2017; Kumar et al., 2022). Earlier studies suggested that certain triggering factors in a susceptible person may stimulate trigeminal nerves of the trigeminovascular system to release glutamate neurotransmitters and select vasoactive neuropeptides (e.g., CGRP, substance P, and neurokinin A) which then contribute to the development of neurogenic inflammation (Brain et al., 1985; Edvinsson et al., 1985; Uddman et al., 1985; Storer et al., 2004; Recober et al., 2009; Erdener and Dalkara, 2014; Russo, 2015a; Russo, 2015b; Jacobs and Dussor, 2016; Tso and Goadsby, 2017; Hendrikse et al., 2018). Among these factors, CGRP appears to be particularly important in the mediation of migraine headaches, perhaps *via* the cAMP-dependent and cAMP-independent pathways (Brain et al., 1985; Edvinsson et al., 1985; Uddman et al., 1985; Storer et al., 2004; Recober et al., 2009; Erdener and Dalkara, 2014; Russo, 2015a; Russo, 2015b; Jacobs and Dussor, 2016; Tso and Goadsby, 2017; Hendrikse et al., 2018). CGRP is considered a key mediator based on the observation that (1) plasma CGRP levels increase in the external jugular vein during migraine attacks and (2) exogenous CGRP increases migraine pain in susceptible patients (Edvinsson, 2008a; Edvinsson, 2008b; Durham and Masterson, 2013; Erdener and Dalkara, 2014; Russo, 2015b; Geppetti et al., 2015; Jacobs and Dussor, 2016). In support of this hypothesis, four anti-CGRP and anti-CGRP receptor antibodies (i.e., galcanezumab, fremanezumab, eptinezumab, and erenumab) have been approved by the FDA to treat chronic migraine since 2018 (Aiyar et al., 2001; Verheggen et al., 2002; Zeller et al., 2008; Mitsikostas and Rapoport, 2015; Shi et al., 2016; Deen et al., 2017; Tso and Goadsby, 2017; Choy, 2018; Tepper, 2018; Al-Hassany et al., 2022). In addition, three small molecule antagonists were recently approved for the prevention and/or treatment of acute migraine (Goadsby et al., 2020; Ailani et al., 2021; Croop et al., 2021; Altamura et al., 2022). In addition to migraine, excessive CGRP release was associated with pain in patients with arthritis, complex regional pain syndrome, and diabetic neuropathy (Schou et al., 2017). Although these advances have improved the care of many patients, it appears that these anti-CGRP therapeutics have limited efficacy in many patients (i.e., a net reduction of two to three headache days in patients who on average have 8–14 migraine headache days per month when compared to the placebo group) (Deen et al., 2017), and headache in these patients remains poorly controlled (Ailani et al., 2021; Al-Hassany et al., 2022; Ornello et al., 2022).

Because antibodies have a low volume of distribution, and mainly act by reducing the circulating level of CGRP and CGRP signaling in cells that are in proximity to vessels (Edvinsson, 2008a; Edvinsson, 2008b; Erdener and Dalkara, 2014; Deen et al., 2017), a long-acting high-potency peptide antagonist could be an alternative candidate for sustained prevention of migraine headache. Such candidates would have a high volume of distribution and better access to neuronal targets. In addition, because the CGRP-related adrenomedullin (ADM) peptide, which mainly signals through the CLR/RAMP2 receptor, has been implicated in the development of inflammatory hyperalgesia and migraine pain and because CGRP and ADM receptors are systematically present in human trigeminal ganglia (Moreno et al., 1999), a peptide antagonist that can target multiple CLR/RAMP receptors may have a better potential to alleviate headaches in patients with severe migraine (Ma et al., 2006; Fernandez et al., 2010; Wang et al., 2011; Zeng et al., 2014; Garelja and Hay, 2022; Rees et al., 2022).

Based on the screening of a series of chimeric ADM/adrenomedullin 2 (ADM2) peptides, we have recently identified a group of potent peptide CLR/RAMP receptor agonists (Chang et al. 2022), and peptide antagonists that potently inhibit CLR/RAMP1 and/or 2 signaling (Chang and Hsu, 2019). By serendipity, we have also found that select antagonists such as ADE651 and ADE609 are capable of self-assembling into hydrogels *in situ* at low concentrations. Because self-assembled peptide hydrogel has been used to deliver the somatostatin receptor agonist, lanreotide Autogel, as a monthly injection to treat acromegaly and neuroendocrine tumors (NETs) (Pouget et al., 2010; Salvatori et al., 2010; Oberg and Lamberts, 2016; Fattah and Brayden, 2017), these gel-forming antagonists may represent promising candidates for sustained inhibition of CGRP- and/or ADM-mediated migraine pain in patients who have not been sufficiently served by existing migraine drugs. In the present study, we used the ADE651 analog as a prototype to characterize the release of antagonist analog gel *in vivo* and the effect on CGRP-induced dermal blood flow in the hindlimbs of rats. Further development of these gel-forming antagonists may provide an alternative strategy to block CLR/RAMP1- and/or 2-mediated chronic migraine headaches in patients.

## Materials and methods

### Chemicals and reagents

Wild-type and palmitoylated analogs of different peptides (purity >95%) were synthesized and characterized by LifeTein LLC (Hillsborough, NJ). The peptides were synthesized on ChemMatrix Rink Amide resin, using standard Fmoc synthesis protocol with DIC/Cl-HOBt coupling, on an APEX 396 automatic synthesizer. The resin was swollen in DMF for 30 min, treated with 20% Piperidine-DMF for 8 min to remove the Fmoc protecting group at 50 °C, and washed with DMF three times. For the coupling reaction, the resin was added with Fmoc-protected amino acid, Cl-HOBt, DIC and NMP. The mixture was vortexed for 20 min at 50°C, followed by washing with DMF. The cycle of deprotection and coupling steps was repeated until the last amino acid residue was assembled. After the final Fmoc protecting group was removed, the resin was treated with 20% acetic anhydride-NMP for 20 min. The resin was washed with DMF, DCM, and dried with air. The peptides were cleaved using a TFA cocktail (95% TFA, 2.5% water, and 2.5% TIS). Crude peptides were precipitated by adding ice-chilled anhydrous ethyl ether, washed with anhydrous ethyl ether three times, and dried. The crude peptide was then prep-HPLC purified.

### Visual and microrheology viscosity assay of gel-forming capability

To evaluate the ability of peptides to form gels *in situ*, we initially determined this tendency qualitatively based on visual examinations of peptide solutions using a tube-tapping method. In addition, we quantitatively determined the viscosity of select peptide solutions using a viscometer (Rheosense Inc; http://www.rheosense.com/products/viscometers) (Yan and Pochan, 2010). In the visual assay, aliquots of peptides were dissolved in an aqueous solution (i.e., de-ionized water, saline, or 5% glucose solution). The peptide that dissolved instantly and stayed as a clear solution without obvious macroscopic change in viscosity at 20 min after mixing was considered soluble. If macroscopic particles of peptides remained in the solution at 20 min after mixing, the peptide was considered insoluble. If the peptide solution exhibited a high viscosity at 20 min after mixing, and the solution conformation only changed slowly when the vial is tilted 90^o^ and tapped with a finger, the analog was considered a gel-forming peptide.

We studied the gel-forming activity of a total of 17 ADE651 variants (ADE651 and variants ADE651A–ADE651P which have unusual amino acid substitution at the junction sequence; Table 1) based on the visual method. The modification in these variants included the substitution of the only leucine with Aib, Ahx, Nva, Cit, Hyp, Nle, Orn, Nal, Abu, Met (O2), Dab, or β-alanine (ADE651A–ADE651K and ADE651P) as well as the substitution of the second lysine with Lys (Me), Arg (Me), Lys (Pyr), or Lys (Mpa) (ADE651L–ADE651O).

**TABLE 1:** The sequence and gel-forming capability of ADE651 and 16 variants as well as the IC_50_ values for antagonizing CGRP-induced CLR/RAMP1 signaling of select ADE651 variants.

Testing articles		Gel-forming ability	IC_50_ (nM)	Maximum response
BIBN4096			0.2	100.5
ADE651	Pal-KVQKLSAPVDPSSPHSY-NH_2_	**Y**	2.2	101.1
ADE651A	Pal-KVQK-Aib-SAPVDPSSPHSY-NH_2_	N		
ADE651B	Pal-KVQK-Ahx-SAPVDPSSPHSY-NH_2_	N		
ADE651C	Pal-KVQK-Nva-SAPVDPSSPHSY-NH_2_	**Y**	1.3	101
ADE651D	Pal-KVQK-Cit-SAPVDPSSPHSY-NH_2_	N		
ADE651E	Pal-KVQK-Hyp-SAPVDPSSPHSY-NH_2_	N		
ADE651F	Pal-KVQK-Nle-SAPVDPSSPHSY-NH_2_	N		
ADE651G	Pal-KVQK-Orn-SAPVDPSSPHSY-NH_2_	N		
ADE651H	Pal-KVQK-Nal-SAPVDPSSPHSY-NH_2_	**Y**	15.1	100.1
ADE651I	Pal-KVQK-Abu-SAPVDPSSPHSY-NH_2_	N		
ADE651J	Pal-KVQK- Met-(O2)-SAPVDPSSPHSY-NH_2_	**Y**	0.9	100.4
ADE651K	Pal-KVQK-DAB-SAPVDPSSPHSY-NH_2_	N		
ADE651L	Pal-KVQ- Lysine (Me)-LSAPVDPSSPHSY-NH_2_	**Y**	2.5	100.2
ADE651M	Pal-KVQ-Arginine (Me)-LSAPVDPSSPHSY-NH_2_	**Y**	2.3	100.7
ADE651N	Pal-KVQ-Lys (Pyr)-LSAPVDPSSPHSY-NH_2_	**Y**	1.1	99.9
ADE651O	Pal-KVQ-Lys (Mpa)-LSAPVDPSSPHSY-NH_2_	**Y**	50.4	99.9
ADE651P	Pal-KVQK-β-alanine-SAPVDPSSPHSY-NH_2_	N		

The gel-forming capability of ADE651 variants was determined based on the visual inspection of 11% peptide solutions. Y: can form a liquid gel; N: lacks the ability to efficiently form a liquid gel. Seven variants, C, H, J, L, M, N, and O, retained the gel-forming capability. All other variants were insoluble or partially soluble at the 11% level.

Pal indicates the N-terminal palmitoylation modification. The antagonistic activity on β-CGRP-mediated CLR/RAMP1 signaling is described as IC_50_ and the maximum response in % of positive control. The potency of a small molecule CGRP antagonist positive control, BIBN4096, is provided for comparison.

In addition, we quantitatively determined the viscosity of ADE651 and control peptides using the Rheosense viscometer. The peptide solution was loaded onto a Rheosense viscometer chip, and the sample was injected at a constant flow rate through the flow channel with multiple pressure sensors that monitor the pressure drop from the inlet to the outlet. Because the pressure drop is correlated with the shear stress at the boundary wall, the instrument determines the rheological properties based on the standard principles of rheometry (Yan and Pochan, 2010).

### Assay of CLR/RAMP1 signaling

The assay of the antagonistic activity of analogs was conducted by the Eurofins Discoverx using the HitHunter cAMP XS + assay as described earlier (Bradley and McLoughlin, 2009; Chang and Hsu, 2019). The dose-dependent inhibitory effect of antagonists was studied in duplicate, at 10 different concentrations. Determination of the half-maximal inhibitory concentration (IC_50_) was performed using a 10-point dose-response curve. The starting concentration was 10 μM, and it was serially diluted 3-fold, in DMSO. Human β-CGRP (Eurofins Discoverx, Fremont, CA) was used as the agonist and a positive control in the assay. The assay of ADE651 has been performed in at least four different separate studies using at least four different batches of peptides. Using the Hit Hunter® assay (CALCRL-RAMP1 cAMP assay in the antagonist mode; https://www.discoverx.com/products/cell-line/cho-k1-calcrl-ramp1-gs-cell-line-95-0164c2), the eight compounds in Table 1 were assayed side-by-side once, and the IC_50_ was derived based on the standard assay of duplicate samples of a ten-point curve. In this homogenous, non-imaging assay, the activation of CLR/RAMP1 signaling was evaluated using the Enzyme Fragment Complementation (EFC) technology with β-galactosidase (β-Gal) as the functional reporter. The enzyme was split into two complementary portions: an enzyme acceptor and an enzyme donor. The enzyme donor is fused to cAMP and it competes with cAMP generated by cells

For binding to a cAMP-specific antibody. Active β-Gal is formed by complementation of exogenous enzyme acceptor to any unbound enzyme donor-cAMP. The active enzyme then converted a chemiluminescent substrate to generate a signal. Briefly, cAMP Hunter cell lines with the select receptor were expanded from freezer stocks (Bradley and McLoughlin, 2009), and cells were seeded in a total volume of 20 μL into 384-well microplates and incubated at 37°C for the appropriate time. For the determination of antagonistic activity, cells were pre-incubated with a sample followed by the CGRP agonist challenge at the EC_80_ concentration. In this assay, the EC_50_ and EC_80_ concentrations for β-CGRP were 0.16 nM and 0.37nM, respectively. Known antagonists, including BIBN4096BS and ADE651, were used as controls. Intermediate dilution of samples was performed to generate 4X sample in the assay buffer. Before the assay, media was aspirated from cells and replaced with 10 μL 1:1 HBSS/Hepes:cAMP XS + Ab reagent, and 5 μL of 4X compound was added to the cells and incubated for 30 min. Then, 5 μL of 4X EC_80_ CGRP agonist was added to cells and incubated at 37°C or room temperature for the appropriate time. Vehicle concentration was 1%.

After compound incubation, the assay signal was generated through incubation with 20 μL cAMP XS + ED/CL lysis cocktail for 1 h followed by incubation with 20 μL cAMP XS + EA reagent for 3 h at room temperature. The chemiluminescent signal was read with a PerkinElmer chemiluminescent plate reader. The compound activity was analyzed using a CBIS data analysis suite (ChemInnovation, CA). The percentage inhibition was calculated using the following formula: % Inhibition = 100% x (1 - (mean RLU of test sample - mean RLU of vehicle control)/(mean RLU of EC_80_ control - mean RLU of vehicle control)). Data were normalized to the maximal and minimal response observed in the presence of EC_80_ agonist and vehicle.

### Measurement of the passage of peptide molecules through Centricon filters

To evaluate whether gel-formation reduces the movement of peptide molecules in solution, we used the Centricon^®^ filters (Millipore) to separate samples and the carriers based on their molecular mass. The sample is monomeric peptides, and the carrier is the self-assembled gel. One milligram aliquots of the peptides were first dissolved as 0.1% solution in 5% glucose, 0.1% solution in saline, 5% solution in 5% glucose, 10% solution in 5% glucose, and 20% solution in 5% glucose. Ten minutes later, the 5%, 10%, and 20% solutions were diluted to the 0.1% level. Aliquots of these solutions were sampled and dispensed into separate Centricon columns (30,000 MW cutoff), and the monomeric peptide and gel molecules were separated by centrifugation for 15 min (2000 x g). The level of peptide in the elutes was determined by specific EIAs (Phoenix Pharmaceuticals Inc.).

### Animals and ethics statement

*In vivo* experiments were conducted using adult male Sprague-Dawley rats (body weight 150–200 g, Charles River) that were 10–14 weeks old. Rats were housed in the Cardio-lab LLC (Gaithersburg, MD) animal care facilities. All procedures were approved by the Institutional Animal Care and Use Committees and all animals were managed in full compliance with the requirements of the Animal Welfare Act and in accordance with the National Institute of Health Guide for the Care and Use of Laboratory Animals. Animals were euthanized by CO_2_ individually at the end of experiments.

### Analysis of the release of ADE651 from gel solution *in vivo*

Single-dose pharmacokinetics of gel solution was investigated in male Sprague-Dawley rats. Rats received a peptide solution *via* the subcutaneous route. Blood samples were collected at pre-dose, 8 h, 1, 2, 4, and 8 days *via* a catheter that has been cannulated to the jugular vein (Charles River), and 0.5–1.0 ml of blood samples were collected at each time point. The animals were individually housed, plasma was obtained by centrifugation, and the peptide level in samples was determined by specific ADM2 EIAs (Catalogue no. EK-010–48, Phoenix Pharmaceuticals Inc.). It has a 0% cross-reactivity to ADM or CGRPs and has a sensitivity of 0.19 ng/ml. The Inter-assay variation was <15%. While the ADM2 EIA recognizes the C-terminal HSY-NH2 motif in the ADM2 and ADE651 peptides in a linear manner, its efficiency for detecting ADE651 was reduced by 2.5-fold, perhaps due to lipidation interference. Therefore, the assay would underestimate the level of ADE651 by 2.5 folds, and the change in ADE651 level is presented as the fold-change of immunoreactive ADM2 level in the pharmacokinetic study.

### Measurements of dermal blood flow and vasodilation in rat hindlimbs

Adult Sprague Dawley male rats (body weight 150–200 g) were randomly divided into treatment groups. Animals were individually housed during the experiment. After acclimation, rats were anesthetized and stabilized under 2.5% isoflurane, and were placed on a heating pad. Testing articles were prepared just before the experiment and individually administered subcutaneously into the hindlimbs. Human β-CGRP (LifeTein LLC, Hillsborough, NJ) was dissolved in a saline solution, and the antagonist solution was prepared in a 5% glucose solution. The CGRP solution was used as a vasodilator to increase dermal blood flow *via* the CLR/RAMP receptors (Buntinx et al., 2015; Vu et al., 2017). In this experiment, the antagonist gel solution was administered at 0 h as a liquid solution shortly after dissolution to prevent issues associated with the formation of semi-solid gel when the peptide is dissolved at high levels. The CGRP solution was injected (1 mg/100 µL) at 4 h after gel administration. The measurement of dermal blood flow continued for 2 h after CGRP administration because the CGRP’s vasodilatory effect lasts only ∼2–3 h. The amount of blood flow was determined by laser Doppler imaging as described earlier (Van der Schueren et al., 2007; Benschop et al., 2014). The laser Doppler scan series of the hindlimbs began with two baseline scans and was followed by two scans each at select time points. Data are reported as percent change from the average baseline scans.

### Statistical analysis

Comparisons among test groups were performed by the ANOVA test followed by Tukey-Kramer or z-test analysis, or Student’s *t*-test using the Excel Analysis ToolPak package. The data were presented as mean ± SEM, and the significance was accepted at *p* < 0.05.

## Results

### Select chimeric CLR/RAMP receptor antagonists self-assemble to form gels *in situ*

**FIGURE 1:**
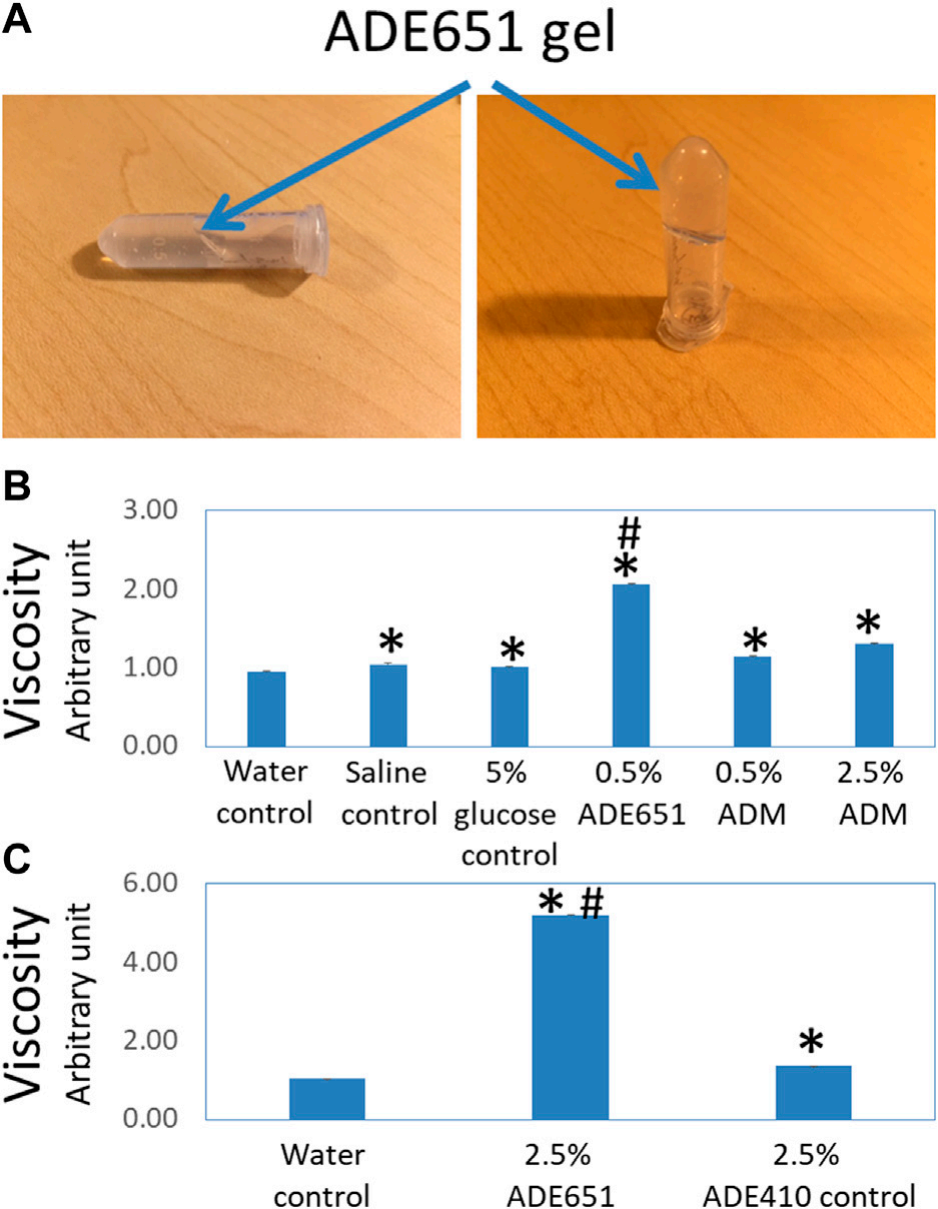
ADE651 forms a liquid gel *in situ*. **(A)** Pictures of an 11% ADE651 solution at 30 min after dissolution. The solution became viscous and lost the ability to flow freely in the tube. The position of the gel mass is indicated by blue arrows. **(B)** The viscosity of peptide solutions (N = 5 per group). The viscosity was determined by a Rheosense viscometer. The solutions included a water control, a saline control, a 5% glucose control, a 0.5% ADE651 in 5% glucose, a 0.5% ADM in 5% glucose, and a 2.5% ADM in 5% glucose. *, Significantly different from the water control. #, Significantly different from the 0.5% and 2.5% ADM solutions. **(C)** The viscosity of 2.5% ADE651 and 2.5% ADE410 control solutions. The ADE410 control peptide shares the C-terminal sequence with ADE651 and its sequence is VGCRFGTCTVQKLAHLWQLMGPAGRQDSAPVDPSSPHSY-NH2. The statistics used included ANOVA and Student’s *t*-test. *, Significantly different from the water control. #, Significantly different from the 2.5% ADE410 solution. Data are mean ± SEM of 5 separate readings.

Based on the analysis of lipidated chimeric peptides, we have previously identified a series of potent peptide antagonists that inhibit CLR/RAMP1 and/or 2. By serendipity, we also found that a 17-amino-acid analog (i.e., ADE651 or Antagonist 2-4 in Chang and Hsu (2019) and a 28-amino-acid (i.e., ADE609 or Antagonist 1-2 in Chang and Hsu (2019) antagonist self-assemble to form liquid gels *in situ* (Chang and Hsu, 2019). Unlike nonlipidated chimeric peptides which dissolved and stayed as a clear liquid solution for days, 6–20% solutions of these peptides became viscous at 20 min after dissolution, and the solution did not flow as a liquid when the vial is tilted 90^o^ and tapped (Figure 1A). Because ADE651 forms gels at a faster pace compared to ADE609, we chose it as a prototype for further characterization.

Studies of the viscosity of peptide solutions with a viscometer showed that the viscosities of the saline solution and 5% glucose solution are significantly higher than that of the water control (Figure 1B). While the viscosity of 0.5% and 2.5% ADM solutions was significantly higher than that of water, the viscosity of these solutions was like that of 5% glucose solution. Consistent with the visual analysis, the viscosity of the 0.5% ADE651 solution was significantly higher than that of all other solutions evaluated (Figure 1B). In a separate experiment, we compared the viscosity of ADE651 to a control chimeric ADM/ADM2 peptide (i.e., ADE410) which does not form a liquid gel at 6–20% level. It showed that the viscosity of 2.5% ADE651 solution is almost 4 times that of the 2.5% ADE410 solution (5.19 ± 0.01 vs 1.36 ± 0.01; Figure 1C). These data suggested that the ADE651 analog has a high propensity to form gels.

### Residues at the junctional region of ADE651 are important for gaining potent antagonistic activity and gel-forming activity

Because ADE651 has a potent antagonistic effect on CGRP signaling and the second lysine residue was shown to be important for maintaining its bioactivity (Chang and Hsu, 2019), we hypothesized that the junctional sequence of this peptide could be important for gaining the gel-forming capability and enhanced antagonistic activity. Accordingly, we studied a total of 16 ADE651 variants (ADE651A–ADE651P) with single residue substitution at the junction of ADM and ADM2 sequences of ADE651 (Table 1). These modifications included the substitution of the only leucine with unusual amino acids including, Aib, Ahx, Nva, Cit, Hyp, Nle, Orn, Nal, Abu, Met (O2), Dab, and β-alanine (ADE651A–ADE651K and ADE651P) as well as the substitution of the second lysine with Lys (Me), Arg (Me), Lys (Pyr), or Lys (Mpa) (ADE651L–ADE651O). Visual analysis of the gel-forming activity of these variants at 11% concentration showed that seven variants, C, H, J, L, M, N, and O, retain the gel-forming capability. All other variants were insoluble or partially soluble at the 11% level. In addition, we measured the antagonistic activity of variants C, H, J, L, M, N, and O toward CGRP-mediated CLR/RAMP1 signaling (Table 1). Although H and O variants had a significantly reduced antagonistic activity compared to ADE651, C, J, L, M, and N variants retained the potent antagonistic activity. These data suggest that a suitable conformation at the junctional region of ADE651 is important for determining the ability to inhibit CGRP signaling and form a liquid gel. Because the antagonistic and gel-forming activities of C, J, L, M, and N variants were not significantly different from that of ADE651, we continued the study with the better-characterized ADE651 peptide.

### Gel-formation slows the passage of ADE651 molecules through the Centricon filter

**FIGURE 2:**
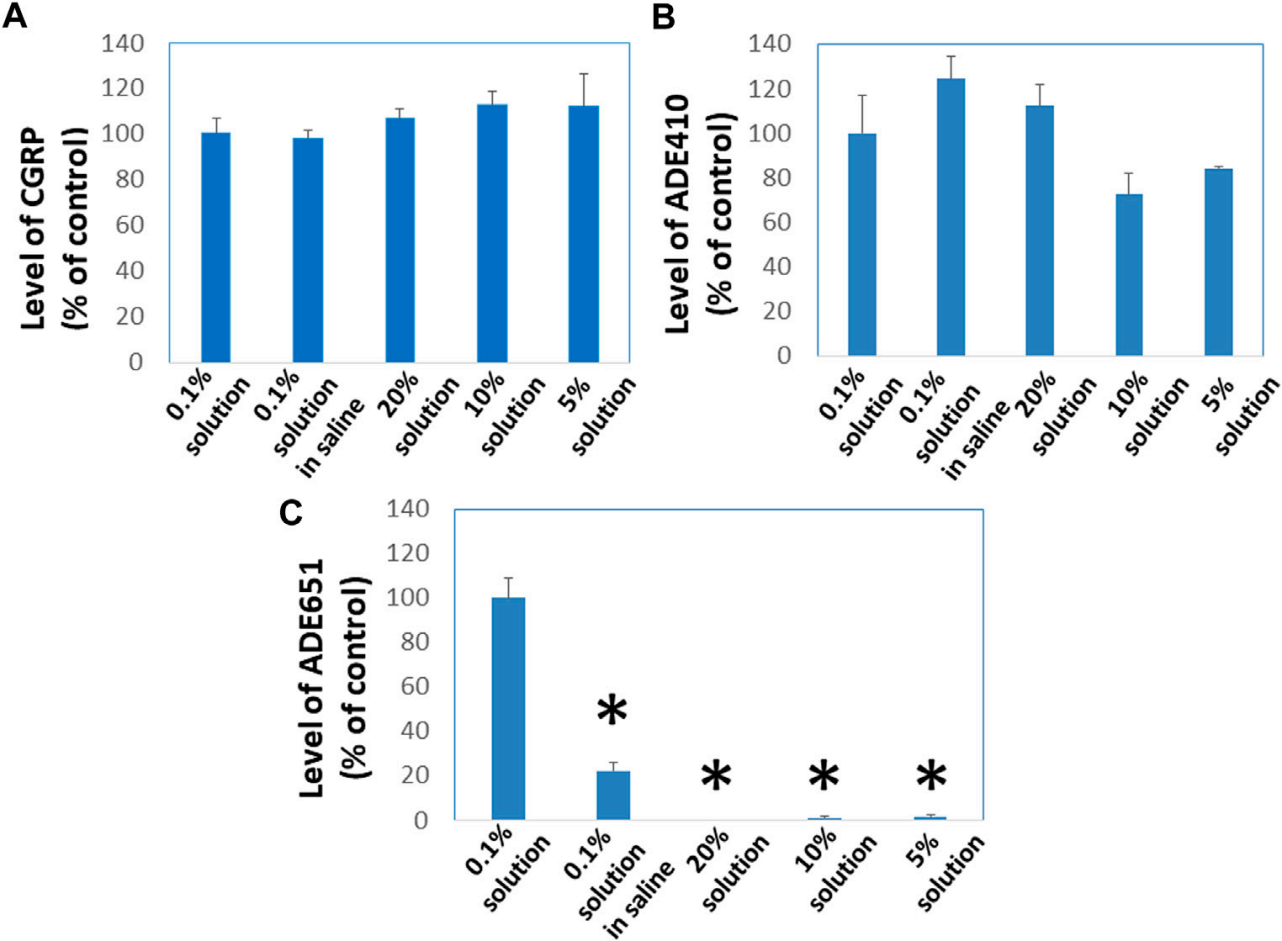
ADE651 forms gels *in situ* and this process impedes the passage of ADE651 molecules through Centricon membrane filters. To determine whether gel-formation reduces the freedom of ADE651 molecules to move in solution, we analyzed the passage of **(A)** CGRP, **(B)** a control peptide ADE410, and **(C)** ADE651 through Centricon membrane filters (MW cutoff: 30,000; N = 3 per group). One milligram aliquots of the peptides were first dissolved as 0.1% solution in 5% glucose (0.1% solution), 0.1% solution in saline, 5% solution in 5% glucose (5% solution), 10% solution in 5% glucose (10% solution) or 20% solution in 5% glucose (20% solution). Ten minutes later, the 5%, 10%, and 20% solutions were diluted to the 0.1% level. All solutions were then dispensed into individual Centricon columns before centrifugation for 15 min. Levels of peptides in the elute were determined by specific CGRP EIA or ADM2 EIA that detects the C-terminal sequence of ADE651 and ADE410. The statistics used included ANOVA and Student’s *t*-test. *, Significantly different from the 0.1% solution control. Data are mean ± SEM of triplicate samples.

To evaluate whether gel-formation slows the movement of ADE651 molecules in solution, we analyzed the ability of ADE651 molecules to pass through Centricon filters at different concentrations. ADE651, CGRP, and the ADE410 control peptides are soluble from 0.1% to 20% in 5% glucose solution. However, ADE651 has low solubility in the saline, and the solution appeared cloudy at 6–20%. Before the filtration with the Centricon filter, aliquots of these peptides were first dissolved at the target concentrations (i.e., 0.1, 5, 10, or 20%), and the high-concentration samples were then diluted to the 0.1% level 10 min later. Analysis of the peptide level in the elutes showed that the level of CGRP is similar in elutes from 0.1, 5, 10, and 20% groups (Figure 2A). Likewise, the level of ADE410 peptide in elutes from different solution groups was similar (Figure 2B). On the other hand, the level of ADE651 in the elute from the 0.1% saline group was significantly lower than that of the 0.1% solution in 5% glucose group, perhaps due to its low solubility in the saline (Figure 2C). While ADE651 was soluble at 5–20% in 5% glucose, the level of ADE651 in the elutes from 5%, 10%, and 20% solution groups was reduced by more than 98% when compared to the 0.1% solution group, suggesting that most ADE651 molecules were strained in the gel and cannot pass through the Centricon filter membrane when it was first dissolved at a high concentration (Figure 2C).

### Subcutaneous administration of ADE651 gel solution leads to the sustained presence of ADE651 in the circulation of rats

**FIGURE 3:**
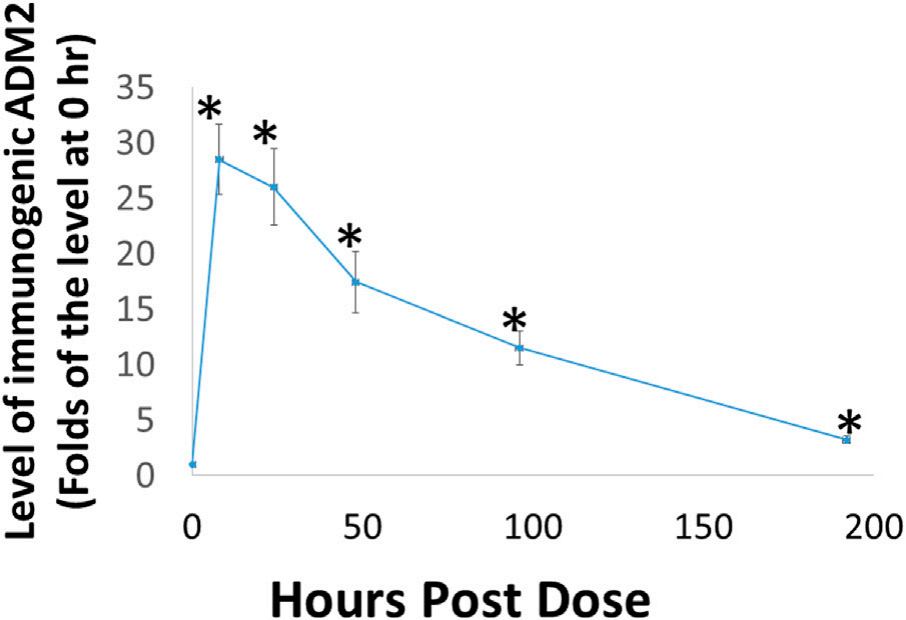
Subcutaneous administration of ADE651 gel solution led to the prolonged presence of the peptide in the circulation of adult Sprague-Dawley rats. Aliquots of ADE651 gel solution (200 µL solution; 32 mg in 16% solution) were delivered subcutaneously to rats, and blood samples were collected at pre-dose, 8, 24, 48, 96, and 192 h after injection. The circulating level of ADE651 in the plasma was significantly increased from eight to 192 h after drug administration. Peptide level was determined by specific ADM2 EIA that detects the C-terminal sequence of ADE651. The statistics used included ANOVA and Student’s *t*-test. *, Significantly different from the control level at 0 h. Data are mean ± SEM of three separate animals.

Because the rate of dissociation of hydrogels depends on electrostatic interactions among the monomeric peptide, solvent, and solutes, the release of ADE651 from the gel is likely affected by peptide concentration and other solutes (Yan and Pochan, 2010; Kopecek and Yang, 2012; Thomas et al., 2016). Therefore, it is important to characterize the release kinetics of ADE651 *in vivo* before studying its efficacy in animals. Analysis of the circulating level of ADE651 in rats after subcutaneous injection of a 16% ADE651 solution showed that the level of ADE651 peaks at 8 h after administration, and the peptide level gradually decreased along the 8-day sampling period (Figure 3). The level of ADE651 remained significantly elevated at 8 days after injection. These data suggest that, unlike the parental ADM and ADM2 peptides which have a short half-life (i.e., <1 h resident time after subcutaneous injection) (Nagata et al., 2022), the gel formulation allows the ADE651 peptide to be slowly released over a week after administration.

### Subcutaneous administration of ADE651 gel solution inhibits CGRP-induced vasodilation in rat hindlimbs

**FIGURE 4:**
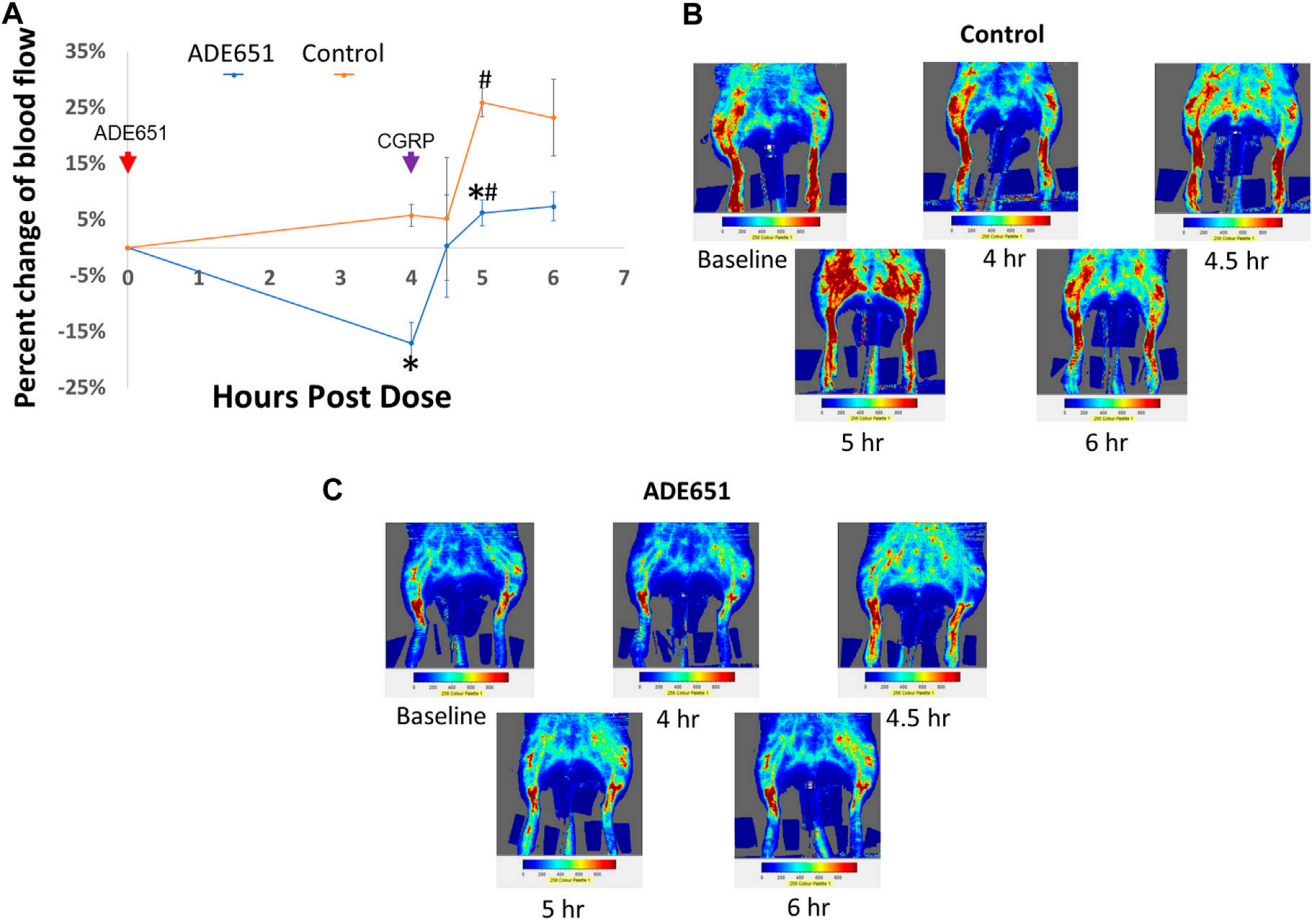
Reduction of CGRP-induced dermal vasodilation in the hindlimbs of adult rats by the ADE651 gel solution. **(A)** Percent change in blood flow from the average of two baseline scans at different time points. The anesthetized Sprague-Dawley rats received a subcutaneous injection of saline (control group) or an ADE651 gel solution (100 µL solution, 8 mg in 8% solution; ADE651 group) in the hindlimb after the basal scans with a laser Doppler imager at the beginning of the experiment (N = 3 per group). At 4 h after the initial injection, an aliquot of vasodilator CGRP (1 mg/100 µL solution) was delivered subcutaneously adjacent to the first injection. Dermal blood flow in the hindlimbs was again scanned at 4, 4.5, 5, and 6 h after the start of the experiment. The time points of drug injection are indicated by red and green arrows. Data are mean ± SEM of three separate animals. *, Significantly different from the control group. #, Significantly different from blood flow that was recorded at the 4 h time point. The statistics used included ANOVA and Student’s *t*-test. **(B)** Representative scans of dermal blood flow at different time points in control animals that received a saline injection at 0 h (i.e., baseline). The CGRP treatment significantly increased blood flow in the hindlimbs of control rats. **(C)** Representative scans of blood flow at different time points in animals that received the ADE651 pretreatment at 0 h. The ADE651 pretreatment significantly reduced the CGRP-induced and normal vasodilation in the hindlimbs.

To evaluate whether ADE651 is capable of blocking CGRP signaling *in vivo*, we studied its effects on CGRP-induced increase of dermal blood flow in the hindlimbs of anesthetized rats. Based on the laser Doppler imaging analysis, we quantified the amount of blood flow in the hindlimbs at different time points after drug treatment. ADE651 or saline solution was administered at 0 h of the experiment, and the vasodilator CGRP was injected at 4 h after the initial treatment. As shown in Figures 4A,B, the injection of CGRP (1 mg/100 µL solution) in control animals at 4 h after the start of the experiment led to significant increases in blood flow in the hindlimbs at the 5 h time point, and the increase was >25%. By contrast, in animals that were pretreated with a subcutaneous injection of ADE651 (8 mg in 8% solution), the CGRP treatment only led to a 6% increase in blood flow at the 5 h time point (Figures 4A,C). In addition, we found that the amount of blood flow in the ADE651 treatment group was significantly lower than that of the control animals before the CGRP treatment (i.e., at the 4 h time point). These data suggested that ADE651 treatment not only reduced the CGRP-induced increase in blood flow but also suppressed the action of endogenous vasodilatory factors in animals.

## Discussion

Although CGRP has emerged as a key target for migraine treatment, many patients have limited responses to existing anti-CGRP therapeutics. As such, there is an emerging need for alternative strategies to target the validated and yet not fully explored CLR/RAMP-mediated pain signaling. Studies of a gel-forming CGRP antagonist showed that the antagonist gel slowly dissociates and exerts a sustained effect on CGRP signaling. Because the self-assembled gel represents an ideal mode for delivering peptide therapeutics, and because the peptide antagonist could have a high volume of distribution and a better chance to target neuronal receptors compared to anti-CGRP antibodies, the gel-forming antagonists may represent promising candidates to treat chronic migraine which cannot be ameliorated by existing drugs. In addition, we speculate that the use of gel-forming dual antagonists such as ADE609 may represent a novel strategy to better serve patients who have a limited response to anti-CGRP drugs by blocking both CGRP- and ADM-mediated pain pathways.

Migraine is a common complex neurovascular disorder that is manifested as episodic and predominantly unilateral throbbing headache with hypersensitivity to light, sound, and movement (Erdener and Dalkara, 2014; Schuster and Rapoport, 2016). According to the World Health Organization, 324 million people worldwide suffer from incapacitating migraine in 2008. In ∼ one-third of patients, migraine headaches are accompanied by an aura involving neurological symptoms such as transient visual, sensory, or motor disturbances (Goadsby et al., 2002). In the US, it was reported that 15% of Americans (9.7% of males and 20.7% of females) had a migraine in the past 3 months (Burch et al., 2021). As such, migraine represents a top global cause of disability-adjusted life years and poses significant costs to society (Steiner et al., 2020; Burch et al., 2021). Prior to the introduction of anti-CGRP therapeutics, migraine is managed with three categories of treatments including, triptans, ergotamines, and analgesics (e.g., non-steroidal anti-inflammatory drugs (NSAID), or the combination of paracetamol, aspirin, and caffeine) (Jackson et al., 2015). These drugs could be effective for symptomatic control if used early. Migraine has also been treated with preventive medications such as topiramate, valproate, angiotensin-converting enzyme inhibitors, Botulinum neurotoxin A (BoNT-A), and angiotensin II receptor antagonists (Halker et al., 2016). However, these medications are inadequate for the control of severe migraine in many patients. Recent progress has led to the approval of several anti-CGRP therapeutics for acute or preventive treatment (Goadsby et al., 2020; Ailani et al., 2021; Croop et al., 2021; Altamura et al., 2022). They include four anti-CGRP and anti-CGRP receptor antibodies (i.e., galcanezumab, fremanezumab, eptinezumab, and erenumab), and three small molecule anti-CGRP therapies. Compared to the placebo group, CGRP antibody therapies resulted in a reduction of monthly migraine days of 1.44–1.55 days (i.e., in patients who on average have 8–14 migraine headache days per month) (Deen et al., 2017; Tso and Goadsby, 2017; Tepper, 2018; Deng et al., 2020; Vandervorst et al., 2021). In addition, eptinezumab was shown to increase the proportion of patients with moderate/maximal optimization from 31% at baseline to 58% at week four compared to 40–50% in the placebo group and was associated with improvements in acute medication optimization compared with placebos (Cady et al., 2022). Furthermore, anti-CGRP antibodies were shown to reduce acute migraine-specific medication days of 1.28 days and the use of acute headache medication (Deng et al., 2020; Tepper et al., 2021). Studies of the economic impact have shown that while anti-CGRP antibodies and Botulinum neurotoxin A have similar prophylactic effects, erenumab had more incremental economic benefits compared to Botulinum neurotoxin A (Chen et al., 2021; Khanal et al., 2022). Moreover, recent studies have shown that the use of galcanezumab improves distress perception in migraine patients’ relatives’ lives (i.e., caregiver relatives’ Stress Scale score) (Fofi et al., 2022). However, the anti-CGRP antibodies’ efficacy is just comparable to those of currently used preventive drugs. For example, the use of topiramate has a reduction of 1.11 migraine days, and this efficacy does not differ from that of anti-CGRP antibodies (Overeem et al., 2021). Nonetheless, anti-CGRP antibodies appeared to have good tolerability and a better efficacy over adverse effect profile compared to traditional therapies. Like anti-CGRP antibodies, the use of small molecule anti-CGRP antagonists resulted in pain reduction. For example, ubrogepant treatment led to pain freedom at 2 h in 21.8% of acute migraine patients compared to 14.3% in the placebo group. The absolute difference in the pain-free rate was 6.4–7.5% (Lipton et al., 2019; Switzer et al., 2022). In the meta-analysis, small molecule antagonists were shown to provide 2 h pain freedom in ∼20% of patients and pain relief in ∼60% (Zhang et al., 2021; Altamura et al., 2022). Because these anti-CGRP therapies have an efficacy comparable to the currently used drugs, migraine headaches remain a life-long burden for many patients (Krymchantowski et al., 2019). New therapeutic approaches that can fully explore the CLR/RAMP-mediated pain pathways may provide an alternative strategy to reduce the medical burden on migraine patients.

Before the introduction of anti-CGRP antibody therapies, the standard-of-care drugs were nonspecific, and the use of these drugs was affected by drug interactions and adverse effect profile (Hepp et al., 2014). On the other hand, the limited efficacy of anti-CGRP antibodies could be associated with the mechanisms of action. Anti-CGRP antibodies block signaling by sequestering the circulating CGRP molecules, and the anti-RAMP1 antibody such as erenumab blocks ligand interactions by projecting a complementary determining region into the interface between CLR and RAMP1 (Garces et al., 2020). Although these antibodies have a high affinity for CGRP or CGRP receptor *in vitro*, drug disposition model analysis showed that antibody concentrations required for effective blocking are relatively high. For example, erenumab concentrations required for 50% and 99% of inhibition of capsaicin-induced dermal blood flow in patients were 0.255 mg/L and 1.134 mg/L, respectively (Vu et al., 2017). Regardless of the mechanism of action, all these antibodies have a large molecule weight and potentially a small volume of distribution, therefore limiting their ability to access target nerves (Al-Hassany et al., 2022; Ornello et al., 2022). They mainly act by reducing the circulating level of CGRP or CGRP signaling in cells that are in proximity of the vascular system (Edvinsson, 2008a; Edvinsson, 2008b; Erdener and Dalkara, 2014; Deen et al., 2017). The volume of distribution reflects how a drug distributes throughout the body. The larger the molecule, the harder it is to passively diffuse out of the vascular compartment. The major determinants of the volume of distribution include molecule size, charge, solubility, pKa, and the lipid/water partition coefficient. For endogenous and exogenous antibodies, the tissue/blood concentration ratio is ∼0.1–0.5 (i.e., antibody concentrations are substantially lower in the interstitial fluid than in the plasma) (Dostalek et al., 2013; Ryman and Meibohm, 2017). At a steady state, antibodies are confined to the vascular and interstitial spaces and have a volume of distribution of 3–8 L (Ovacik and Lin, 2018). For example, the ant-CGRP receptor antibody erenumab has a molecular mass of ∼150 kDa and a volume of distribution of 3.86L, and the galcanezumab has a volume of distribution of 7.3L (https://go.drugbank.com/drugs). On the other hand, peptide drugs generally have a larger volume of distribution compared to antibodies. For example, the volume of distribution of approved peptide therapeutics such as exenatide, abaloparatide, histrelin, octreotide, and setmelanotide was 28.3L, 50.0L, 58.4L, 18.1–30.4L, and 48.7L, respectively. Therefore, small peptide antagonists such as ADE651 and ADE609 may have better access to potential CLR/RAMP targets compared to therapeutic antibodies. Future development of these analog gels may thus provide an alternative strategy to better access the migraine-associated CLR/RAMP receptor targets.

In addition to providing relief for acute migraine, small molecule anti-CGRP therapies such as atogepant and rimegepant, which are referred to as gepant compounds, are approved for preventive treatment (Altamura et al., 2022; Switzer et al., 2022). It is known that anti-CGRP gepants could occupy a binding site close to the interface of the N-terminal domains of CLR and RAMP1, and act by allosteric regulation (Miller et al., 2010). Although the gepants compounds have low IC_50_ values in receptor activation assays *in vitro*, it is well documented that gepants need high concentrations to block CGRP signaling *in vivo*. For example, the EC (90) for telcagepant to inhibit the capsaicin-induced increase in dermal blood flow was ∼909 nM (Sinclair et al., 2010). Therefore, the limited efficacy of small molecule antagonists could be due to restricted bioavailability in target tissues or their specificity for select signaling states (Goadsby et al., 2020; Ailani et al., 2021; Croop et al., 2021; Altamura et al., 2022; Tanna et al., 2022). Because the gel-forming peptide antagonists are expected to block receptor activation *via* a distinct and larger interface compared to small molecule antagonists (Lee et al., 2016), we speculate that the gel-forming antagonists could have a pharmacodynamic characteristic distinct from that of gepant therapies.

In the present study, we chose the ADE651 analog as a prototype because it appeared to have a better gel-forming capability. ADE651 is a 17-amino-acid chimera with 5 N-terminal residues from ADM and 12 C-terminal residues from ADM2 (Chang and Hsu, 2019). Because earlier studies showed that the substitution of ADE651s second lysine with asparagine abolishes its bioactivity, we reasoned that its potent antagonistic and gel-forming activities could be associated with the sequence at the chimeric junction. Consistent with the hypothesis, substitution of the leucine residue at the junction with various unusual amino acids led to a disruption of the ability to form gels. Among the 12 variants with substitution at the leucine position, only three retained the gel-forming activity. Among these three variants, one has reduced antagonistic activity. On the other hand, the substitution of the second lysine with methyl lysine, methyl arginine, Lys-Pyr, or Lys-Mpa has a minimal effect on gel formation. However, modification of the lysine residue with a thiol group-containing Mpa increased the IC_50_ by > 10 folds. These data suggested electrostatic interactions surrounding the junctional sequence could be important for gaining the gel-forming activity and potent antagonistic activity. Further exploration of variants with modifications at these key positions may generate analogs with even better physical-chemical properties (i.e., improved gel-forming capability and stability *in vivo*) compared to analogs studied here.

CGRP family peptides include α- and β-CGRP peptides, ADM, and ADM2 (Kitamura et al., 1994; Michibata et al., 1998; Hinson et al., 2000; Roh et al., 2004; Takei et al., 2004). These peptides signal through CLR/RAMP receptor complexes composed of two transmembrane components, the CLR and one of the three RAMPs (RAMP1, 2, and 3) (McLatchie et al., 1998; Hinson et al., 2000; Roh et al., 2004; Takei et al., 2004; Bell and McDermott, 2008). Whereas CGRPs mainly act through CLR/RAMP1, ADM has a high affinity for CLR/RAMP2 and 3 (McLatchie et al., 1998; Muff et al., 1998). On the other hand, ADM2 is a mild ligand for all three receptors. In addition to CGRP, ADM has been implicated in the regulation of inflammatory heat hyperalgesia, the development of morphine tolerance, and spinal glial activation as well as migraine pain (Ma et al., 2006; Fernandez et al., 2010; Wang et al., 2011; Zeng et al., 2014; Ghanizada et al., 2021; Garelja and Hay, 2022; Rees et al., 2022). Migraine patients infused with ADM developed migraine attacks, suggesting that the ADM-mediated CLR/RAMP2 signaling represents a potential target for migraine treatment (Ghanizada et al., 2021). In a recent study, we reported that palmitoylated ADM/ADM2 chimeras of 27- to 31-amino-acid have potent antagonistic activity toward CLR/RAMP1 and 2. The IC_50_ values of these analogs for CLR/RAMP1 were 10-fold lower than that of CGRP8-37, and the IC_50_ values of them for CLR/RAMP2 were five- to 50-fold lower than that of ADM22-52 (Chang and Hsu, 2019). Although this study has focused on the ADE651 analog, future exploration of gel-forming antagonists that have potent inhibitory activity on multiple CLR/RAMP receptors may generate candidates that can block not only CGRP- but also ADM-mediated pain pathways, therefore providing therapeutic effects not attainable with existing anti-CGRP therapeutics. It is also important to note that the characterization of antagonistic analogs is limited to CLR/RAMP1 and 2 signalings (Chang and Hsu, 2019); future studies that encompass CLR/RAMP3 and the calcitonin receptor (CTR)/RAMP receptor pathways using the same mode of second messenger measurements are needed to fully understand the molecular mechanisms underlying their actions *in vivo* and reveal whether these antagonist gels have unforeseen risks.

Our screening of long-acting antagonists has originally focused on lipidated peptides because lipidation is known to increase the half-lives of various peptides by enabling them to be bound by albumin and other serum proteins, therefore reducing elimination by kidneys and degradation by serum proteases (Drucker et al., 2010). Of interest, we also found that palmitoylation can significantly enhance the bioactivity of various chimeric ADM/ADM2 agonists and antagonists (Chang and Hsu, 2019). This finding is consistent with earlier studies of benzoylated and lipidated CGRP8-37 analogs which have enhanced antagonistic activity, perhaps due to strengthened interactions between the ligand and target cells (Smith et al., 2003; Williams et al., 2018; Jamaluddin et al., 2022). Therefore, a lipidated antagonist such as ADE651 may acquire the long resident time *in vivo* partly *via* an improved interaction with receptors or the cell surface membrane environment (Nanga et al., 2011; Perez-Castells et al., 2012; Russell et al., 2014; Ueda et al., 2019). Although ADE651 and related antagonists are expected to have an extended half-life compared to classical antagonists such as CGRP8-37, the pharmacological characteristics are not sufficient to provide a comparative advantage when compared to anti-CGRP antibodies because the half-life of a lipidated peptide is still limited. Importantly, we found that subcutaneous injection of ADE651 leads to the sustained presence of this peptide in the circulation for >1 week. One of the best-characterized gel-forming peptides is the FDA-approved somatostatin receptor agonist lanreotide (Oberg and Lamberts, 2016). Lanreotide is administered as a 25% gel depot, and the gel slowly releases the lanreotide monomer over a period of a month. This formulation is advantageous because it has an ∼100% loading capacity and the only gel-forming molecule in the gel is the peptide therapeutics itself (Vaishya et al., 2015). However, this triumph has not been translated into other therapeutics because it is not clear how the gel-forming capability can be efficiently introduced into peptides (Grimaldi et al., 2022). Through the study of ADE651, it became clear that advantages provided by the self-assembled gel formulation may allow ADE651 and related antagonists to become viable drug candidates. However, it is important to note that the ability of peptides to form nanostructured gels is governed by multiple forces, including hydrogen bonds, hydrophobic interactions, and π-π aromatic interactions among side chains of amino acids (Yan and Pochan, 2010; Kopecek and Yang, 2012; Thomas et al., 2016). The concentration of peptide and other solutes can significantly affect electrostatic interactions among residues, salt, and water molecules, and hence the strength of gels (Yan and Pochan, 2010; Kopecek and Yang, 2012; Thomas et al., 2016). Therefore, future studies of gel-formation with different adjuvants are critical to revealing whether these analogs can be further improved to provide an even longer period of release *in vivo*.

Because vasodilation models have been used as a biologically relevant way to investigate the anti-CGRP activity of diverse compounds (Brain et al., 1985; Hershey et al., 2005; Van der Schueren et al., 2007; Sinclair et al., 2010; Benschop et al., 2014), we studied ADE651's bioactivity *in vivo* based on its effect on CGRP-induced vasodilation. Although the model showed that ADE651 can block CGRP-induced vasodilation at least 5 h after injection, the pharmacokinetic study indicated that ADE651 gel may exert bioactivity for >1 week after a single injection. Additional vasodilation studies that span a longer period are needed to reveal how long the ADE651's antagonistic activity can last after an injection. Furthermore, studies of the effects of antagonist gels in other migraine models such as the nitroglycerin-provoked hyperalgesia attack and the plantar and orofacial formalin tests are needed to better understand the pharmacodynamic properties of the antagonist gel (Dong et al., 2015; Greco et al., 2015; Sufka et al., 2016).

Other than the regulation of pain perception, CGRP family peptides are important regulators of vascular endothelial functions as well as angiogenesis and lymphangiogenesis (Caron and Smithies, 2001; Shindo et al., 2001; Ichikawa-Shindo et al., 2008; Koyama et al., 2013; Shindo et al., 2013; Koyama et al., 2015). CGRP, ADM, and ADM2 have been shown to promote vascular development and tumor growth (Toda et al., 2008; Smith et al., 2009; Zheng et al., 2010; Mishima et al., 2011; Aslam et al., 2012; Hong et al., 2012; Kurashige et al., 2014; Russell et al., 2014; McIlvried et al., 2022). In tumor-bearing CGRP knockout mice, there is a significant increase in tumor-infiltrating T cells and a significant reduction in tumor size compared to wild-type mice (McIlvried et al., 2022). In addition, many studies have shown that (1) ADM promotes tumor angiogenesis and lymphangiogenesis, (2) the expression of ADM in tumors is associated with the aggressiveness of tumors, distant metastasis, and poor patient prognosis, and (3) blockage of ADM signaling reduces tumor-associated angiogenesis and metastasis of tumor xenografts (Ouafik et al., 2002; Ishikawa et al., 2003; Boudouresque et al., 2005; Nikitenko et al., 2006; Fritz-Six et al., 2008; Jin et al., 2008; Toda et al., 2008; Kaafarani et al., 2009; Tsuchiya et al., 2010; Berenguer-Daize et al., 2013; Karpinich et al., 2013; Liu et al., 2013; Nouguerede et al., 2013; Siclari et al., 2014; Wang et al., 2014; Khalfaoui-Bendriss et al., 2015; Zhou et al., 2015). Therefore, an antagonist gel that inhibits both CLR/RAMP1 and 2 may be useful for blocking CGRP- and ADM-mediated tumor growth and metastasis in patients (Vazquez et al., 2020).

Finally, it is important to note that the existing EIA assay for estimating the ADE651 level is not ideal. It underestimated the level of ADE651 when compared to wild-type ADM2; therefore, future development of a more sensitive assay or protocol to estimate the ADE651 level is needed to precisely characterize the pharmacokinetics of antagonist gels in animals and humans. Moreover, in addition to the identification of gel-forming peptide antagonists described here, we have recently reported the identification of gel-forming peptide agonists for CLR/RAMP receptors (Chang et al. 2022). In that study, we showed that administration of gel-forming agonists results in a sustained increase of dermal blood flow in rats, and the gel formulation allows a localized stimulatory effect. It is conceivable that the administration of gel-forming antagonists could have a similar localized effect. This property could have pros and cons in the future application of these antagonists. It may allow localized blockage of CLR/RAMP signaling; however, for systemic applications, it may result in an adverse effect in local tissues. Future toxicological studies on these issues are critical to evaluate the translational potential of these novel antagonists.

## Conclusion

Based on the analysis of a prototypic gel-forming peptide antagonist, we demonstrated that the antagonist gel allows the antagonist to be slowly released and inhibits CGRP-induced vasodilation. Because the peptide antagonist could have a high volume of distribution and act by blocking receptors on the cell surface, the antagonist gel may represent a novel strategy to ameliorate the CGRP- and/or ADM-provoked migraine headaches in patients.

## Data Availability

The original contributions presented in the study are included in the article/supplementary material further inquiries can be directed to the corresponding author.
